# Sleep deprivation among adolescents in urban and indigenous-rural Mexican communities

**DOI:** 10.1038/s41598-023-28330-8

**Published:** 2023-01-19

**Authors:** Andrea Silva-Caballero, Helen L. Ball, Karen L. Kramer, Gillian R. Bentley

**Affiliations:** 1 Department of Anthropology, Durham University, Durham, DH1 3LE UK; 2 Department of Anthropology, Univesity of Utah, RM 4625, Salt Lake City, USA

**Keywords:** Behavioural ecology, Biological anthropology, Evolutionary developmental biology, Paediatric research

## Abstract

Comparing the nature of adolescent sleep across urban and more isolated, rural settings through an ecological, cross-cultural perspective represents one way to inform sleep nuances and broaden our understanding of human development, wellbeing and evolution. Here we tested the Social Jetlag Hypothesis, according to which contemporary, urban lifestyles and technological advances are associated with sleep insufficiency in adolescents. We documented the adolescent sleep duration (11–16 years old; X̅ = 13.7 ± 1.21; n = 145) in two small agricultural, indigenous and one densely urban context in Mexico to investigate whether adolescents in socio-ecologically distinct locations experience sleep deprivation. Sleep data was assembled with actigraphy, sleep diaries and standardized questionnaires. We employed multilevel models to analyze how distinct biological and socio-cultural factors (i.e., pubertal maturation, chronotype, napping, gender, working/schooling, access to screen-based devices, exposure to light, and social sleep practices) shape adolescent sleep duration. Results suggest that the prevalence of adolescent short sleep quotas is similar in rural, more traditional environments compared to highly urbanized societies, and highlight the influence of social activities on the expression of human sleep. This study challenges current assumptions about natural sleep and how adolescents slept before contemporary technological changes occurred.

## Introduction

Since the late 1970s, sleep researchers have warned against short sleep quotas among adolescents and their negative short- and long-term effects on mental and physical health^1,2^. These concerns led to the formulation of the Social Jetlag Hypothesis (SJH) in 2006, pointing to a shortening in sleep durations due to a misalignment between the endogenous circadian clock (i.e., the internal process regulating the sleep–wake cycles around every 24 h), and the social clock (also known as social zeitgebers) which is dependent on school schedules, social activities and individual choice^3–8^. This misalignment is hypothesized to be particularly acute in industrial/post-industrial societies, characterized by access to electric lights that enable continued activity until late night, as well as by its sedentary, indoors lifestyle, shielded from environmental changes relative to temperature, humidity, noise, wind and moon/sun light^9^. Nonetheless, as long as optimal sleep quotas remain uncertain, the extent of sleep loss and its impact on adolescent wellbeing are still unclear^4,10^. Re-examining the nature of adolescent sleep outside industrialized urban settings through a cross-cultural perspective represents one way to better inform sleep nuances^11,12^, and therefore, broaden our understanding of human development, wellbeing and evolution.

According to existing evidence, normal sleep total duration ranges between 8.5 and 10 h in children (5 years old onwards)^6,13,14^ and between 7 and 9 h in adults (18–40 years old)^15,16^. Notably, adolescents sleep as much as children on their free days (also referred as non-school days), when they are not constrained by school timetables start times, suggesting that their sleep requirements do not change until they become young adults^16^. This seems counterintuitive, since, on the other hand, on school nights adolescents typically express a progressive shortening of their sleep by up to 2–3 h^4–6^, a debt for which they compensate across weekends^17^. This shortening is driven, not by lesser sleep requirements, but by convergent biological, psychological and socio-cultural factors, including school start times^4^.

“Modern”, contemporary urban adolescents, who are oriented towards a late chronotype (i.e., individuals who have strong preferences for extreme late sleep–wake schedules, with later diurnal peaks of alertness and later sleep onsets compared to early and midrange chronotypes^18^) and who must comply with early-morning schooling schedules, are especially vulnerable to sleep deprivation^4^. This vulnerability grows amidst an increasingly hierarchical and complex social life that poses great demands in terms of time, energy and attention^19^, and is further underpinned by ready access to screen-based electronic media devices^17^. In addition, during adolescence parents stop setting bedtimes, and teenagers gain autonomy over their own sleep times^4^. Surprisingly, Gradisar et al.^5^ suggest that sleep deprivation can be aggravated by cultural factors even within such urban, highly industrialized societies. After performing a meta-analysis of 41 surveys addressing adolescent sleep patterns worldwide, they found that South-East Asian adolescents have a later sleep onset than USA and European adolescents, obtaining less sleep and reporting higher rates of sleepiness^5^.

Although it is hypothesized that teenagers in more “traditional”, rural settings (i.e., small-scale societies that are relatively isolated geographically, socially and/or culturally, without ready access to electricity or electronic devices^20–24^) have sleep–wake cycles that are more entrained with natural exposure to light and are able to sleep longer in compliance with their “biological/natural” sleep requirements^25,26^, little is known about normal adolescent sleep in these environments. Previous research performed in rural and/or non-industrial populations in Germany, China, Uganda, Egypt, Brazil and Argentina reveal a wide variation in the sleep duration of adolescents, from 7 to 10.7 h/day^12,25–32^, sometimes below the sleep quota recommended by specialists and clinicians based on studies conducted in U.S.A populations (8.5–10 h/day)^6,13,14^. This observed variability in sleep was explained by several factors, such as aging, pubertal stages, weekday schedules, school workloads, daily and pre-sleep activities, access to electricity, seasonal variations in daylight and bed-sharing habits. Notably, results from these studies are difficult to compare due to differences in length of the studies, age groups, instruments and definitions employed for calculating sleep duration.

Using biosocial, cross-cultural, anthropological perspectives to re-examine current assumptions of adolescent “natural” sleep, the paper here is oriented to better understand the nuances of human sleep behavior. Specifically, we address adolescent sleep in one post-industrial society and two agricultural indigenous communities in Mexico, ranging from dense urban areas with artificial outdoor and indoor lighting, access to the internet, and screen-based device use to a small, rural village with scarce artificial lighting, shared sleeping quarters, no internet, and no device use. The main objectives of this study were to: (1) test the SJH by examining whether adolescents in rural agricultural societies experience sleep deprivation, and (2) investigate what bio-socio-cultural factors influence sleep duration in the three different societies that were studied. Namely, we assessed how working/schooling, daily exposure to natural light, access to electric light and electronic devices, and social sleep practices might alter the duration of adolescent sleep. Building on the SJH, we predicted that: (a) adolescents in rural agricultural settings would suffer less sleep deprivation than urban teenagers, (b) nightly ambient light would shorten sleep duration consistently across sites, and (c) napping rates would be higher in rural conditions but inhibited by urban lifestyles^22,33^. Acknowledging that there is no agreement on the changes that mark the beginning, middle and end of adolescence^34^, we discuss changes in adolescent sleep duration using pubertal stages as reference points.

## Results

### Ecology of adolescent sleep

In terms of access to technology and sleep environments, the three study locations mirror a gradient between highly urban and more rural, subsistence-based societies (Table 1). The three groups are: (1) urban mestizo school teenagers attending a private (fee-paying) school in Mexico City; (2) Totonac agriculturalists from a town in Puebla in east-central Mexico; and (3) Maya agriculturalists from a small village in a more isolated rural area of Campeche in southeast Mexico (Table 2). In Mexico City, all participants have cell/mobile phone coverage as well as broadband at home. In Puebla, they have limited mobile coverage but no broadband. In Campeche, where blackouts were frequent because of heavy rains, teenagers have no broadband nor mobile coverage.

**Table 1:** Micro- and Macro-ecological characteristics of participants' sleep by sites.

Sleep ecology	%	Mexico city	Puebla	Campeche
Access to screen-based devices†	Mobile phones	94	67	57
	Tablets	54	35	9
	Computers	94	18	9
	TV	100	96	98
Darkness/light	Dark	76	39	68
	External illumination ††	18	24	16
	Indoors light	6	37	16
Social sleep	Solitary sleep	72	33	11
	Social sleep	28	67	89
Materiality of sleep	Sleeping surface	Bed	Bed or traditional wooden surface	Hammock
	Housing materials	Concrete, steel, and bricks	Concrete, steel, bricks, rocks, wood, and cardboard	Concrete, steel, bricks, raw earth, palm, wood and cardboard
School demands	School day start	7:30 h	8:00 h	8:00 h
	Commuting times	5–50 min*	5–10 min**	5–10 min**

Data are represented as percentages relative to each sample group.

^†^Unlike urban participants, most Totonac and Maya adolescents from Puebla and Campeche do not personally own screen-based devices but borrow them from a family member. Consequently, their access to such devices is more restricted than participants from Mexico City.

^††^Streetlights or moonlight are considered as external sources of night lighting.

*By walk, car, public transport, or school bus.

**By walk.

**Table 2:** Characteristics of the study sites.

Study site	Geographic location	Economy	Inhabitants and ethnic composition	Main language
Mexico city (south and northeast urban areas)	The city is located in a highlands plateau, 2303 m above sea-level	Urban, postindustrial city with a predominantly service-based economy. Participants belong to relatively affluent and educated families with occupations comprising trades (such as driver, shopkeeper or merchant) to professions (e.g., accountant, engineer, teacher, doctor, dentist) among others	~ 8.9 million inhabitants Mexico City has an admix ethnic background composition, with 89% identifying as "mestizos", 9% belonging to an indigenous group and 2% as African-descendants	Spanish
San Juan Ozelonacaxtla, Huehuetla, Puebla	The town lies 834 m above sea-level at the intersection of two mountain systems	Agricultural Totonac town. By the end of the 1980s electricity and running water were introduced, followed by better roads, schools and a local health center. Since regional orographic characteristics prevented the adoption of mechanized agriculture, traditional farming techniques have prevailed. Household economies mainly depend on agriculture (typically coffee, pepper and corn) and animal husbandry (chicken, ducks, pigs and cattle), but also trades (such as shopkeeper or baker) and remittances from migrant family members working in Mexico City or Puebla de Zaragoza in construction or domestic work. Generally, children and adolescents with migrant parents stayed in the community under the care of close relatives	~ 1368 inhabitants People in San Juan Ozelonacaxtla identify as Totonac, Mexico's tenth largest ethnic group	Highland Totonaku
Xculoc, Hopelchen, Campeche	The village is located in the Yucatan Peninsula plain, 69 m above sea level	Small farming Maya village undergoing demographic and economic transitions with the recent introduction of running water, electricity, new farming techniques for intensive agriculture, schools and roads. Still, the site remains isolated from public transport. Household economies largely depend on subsistence agriculture and crop sales (such as peanuts, corn and squash), animal husbandry (ducks, chicken and sheep), apiculture, construction labor, or work in in neighboring village restaurants	~ 835 inhabitants Population in Xculoc identify as "Mayeros" or Maya, Mexico's second largest ethnic group	Peninsular Maya

Remarkably, a high proportion of rural adolescents in both Puebla and Campeche (47%) report sleeping either with the lights on or being illuminated at night by streetlights or moonlight compared to urban participants in Mexico City (12%). Particularly, 37% of the Totonac participants in Puebla indicate they keep a light on while sleeping, and 24% have an external illumination source. When questioned about the rationale for using an illumination source while sleeping, adolescents indicate that they, or a household member, are afraid of the dark or need to keep their screen-based devices on because watching images help them to fall asleep.

Regarding sleeping spaces, urban participants in Mexico City sleep in areas designed for buffering external sound, wind, light, and temperature oscillations, while rural adolescents sleep in spaces that provide a less effective buffering from external cues. While participants in Mexico City sleep in cement roofed rooms, enclosed by curtains, glass windows, and robust, sealed doors, Totonac teenagers in Puebla sleep under roofs made of baked tiles, corrugated metal, cardboard or cement, without windows and entrances being necessarily glazed or fully sealed. Similarly, Maya teenagers in Campeche sleep in 1-room houses with roofs made of corrugated metal, cardboard or palm leaves, having few curtains, windows or sealed doorways.

It should be noted that the gradient between “modern” postindustrial societies and “traditional” subsistence-based communities is also observable in school demands, a major component of adolescents’ sleep macroecology. In this respect, Mexican rural education varies greatly depending on the local State/Municipal resources and the communities’ expectations and restraints^35,36^. Therefore, lax schooling is observed in Campeche, more structured teaching is found in Puebla, and a fairly rigid structured schooling system operates in Mexico City, where students must meet strict schedules and demanding workloads. Finally, while classes in rural locations begin at 8:00, those in Mexico City start at 7:30. Additionally, it takes 5–50 min to go to school in Mexico City, whether by foot, automobile, public transportation, or school bus. Participants in Puebla and Campeche walk 5–10 min from their houses to their school.

### Duration and (in)sufficiency of sleep

Sleep characteristics were computed for each location (see Methods below) (Table 3a). Participants from Mexico City express the shortest nightly sleep duration on school nights (471 min, SD = 58 min), while Maya participants from Campeche have the shortest sleep quotas on free nights (535 min, SD = 89 min). Respecting daytime sleep, the highest nap ratios are observed among Maya teenagers during both school days and free days (20.1, SD = 27.43 and 18.75, SD = 26.8, respectively).

**Table 3:** (a) Values for sleep timing, duration and daytime napping by sites. Data are represented in minutes as mean (standard deviation). Standard deviation is expressed in minutes for start times, end times and sleep values. (b) Definitions of sleep measurements.

(a)	Weeknight	Site	Start time	End time	Sleep duration	Nap ratio
	School days	Mexico City	22:23 (54.58)	6:12 (27.12)	471.01 (57.94)	17.23 (22.04)
		Puebla	22:31 (65.94)	6:56 (28.45)	508.41 (65.36)	13.07 (18.05)
		Campeche	22:48 (47.33)	6:56 (29.56)	488.66 (50.18)	20.12 (27.43)
	Free nights	Mexico City	23:30 (82.11)	8:45 (93.61)	556.47 (91.39)	14.78 (19.78)
		Puebla	22:33 (73.3)	8:01 (74.99)	571.06 (88.16)	13.56 (19.79)
		Campeche	23:05 (80.01)	7:59 (65.58)	535.39 (89.16)	18.75 (26.85)

(b)	Sleep variable	Definition				
	Sleep duration	The interval between the participant goes to sleep, and the time she/he gets out of bed/hammock/sleeping surface. We scored a period of inactivity in our actigraphy data as sleeping time if it exceeded 210 min				
	Total sleep duration	The total amount of time spent lying in bed/hammock/sleeping surface per day, including daytime naps				
	Sleep start and sleep end	The clock time when time in bed/hammock/sleeping surface starts and when the final morning awakening takes place, respectively. Reported as HH: MM				
	Sleep deprivation	Sleep of shorter duration than the basal need per night of 9–10 h in early adolescence. The term denotes an inadequate quantity of sleep per night that can be acute or chronic (persistent for at least 3 months)				
	Sleep curtailment	Shortened sleep relative to average sleep duration during free/non-work days (when individuals are presumably free to meet their sleep needs). The extent of sleep debt is conventionally calculated through subtracting total sleep duration (TSD) on workdays from the TSD on free days				
	Short sleep	General term describing sleep quotas in early adolescents lower than 9–10 h. This concept encompasses individuals who function well during waking life with little sleep and those who do not and are, therefore, sleep deprived				
	Nap	Period of sleep typically occurring during daylight outside a main big sleep bout (a sleeping period that exceeds at least 210 min). We scored a period of inactivity in our actigraphy data as a nap episode if it fell within the threshold of 15 to 210 min and occurred at least an hour before or after a main big sleep				
	Nap ratio	The proportion of actigraphy-assessed nap days, calculated as the total number of days with at least one observed nap divided by the number of days with actigraphy data. Formula: nap ratio = (number of naps/number of act data) * 100				

The values for overall sleep duration are significantly different between school and free nights (*t* = − 14.6, df = 1,012.9, *p* < 2.2e−16). Likewise, we found significant differences between values for participants’ sleep duration and total sleep duration (TSD) on school days and free days (*t* = − 7.8, df = 605, *p* < 2.2e−16 and *t* = − 7.3, df = 585, *p* < 0.001, each). Additionally, sleep duration values across sites during both, school and non-school days, are significantly different (*F*(2, 603) = 20.5, *p* = 2.38e−09 and *F*(2, 583) = 6.8, *p* = 0.0012, respectively). We found identical statistical differences across sites for TSD values (Table 4).

**Table 4:** Comparison of total sleep duration (TSD) and dimensions of sleep insufficiency between post-industrial urban (Mexico City), and rural agricultural Totonac (Puebla) and Maya (Campeche) adolescent populations.

Duration and (in)sufficiency of sleep		Mexico city †	Puebla	Campeche	Significance
Dimensions of sleep insufficiency					
(a) Short sleep	School night	459.42 (39.3)	490.98 (33.69) ***	485.8 (33.2) ***	F = 9.51, *p* < 0.001
	%	94	78	98	
	Free night	493.5 (37.15)	506.25 (24.63) *	489.19 (40.46)	F = 2.82, *p* = 0.07
	%	29	26	49	
(b) Sleep curtailment	Extent	− 95.9 (59.72)	− 79.12 (46.89) *	− 70.48 (44.63) *	F = 3.76, *p* = 0.03
	%	96	87	88	
(c) Drowsiness	% ≤ 1 time per week	20	55	42	
	% ≥ 1 times per week	80	45	58	
Total sleep duration	School day	478.06 (57.83)	518.5 (69.44)	497.4 (51.19)	F = 20.52, *p* < .001
	Free day	561.99 (92.56)	580.23 (84.91)	550.29 (89.38)	F = 6.8, *p* = .001
	Significance	*t* = − 6.5, *p* < .001	*t* = − 8.1, *p* < .001	*t* = − 9.4, *p* < .001	

Data are represented in minutes as mean (standard deviation). (a) Prevalence and comparison of short sleep quotas (< 9 h/night) on school nights and free nights among the three study sites. (b) Prevalence, extent, and comparison of sleep curtailment values (calculated through the subtraction of the TSD on school days from the TSD on free days). Sleep curtailment can be expressed as a negative value when TSD is shorter during school days compared to free nights, a 0 value when equal, and a positive value when greater. c) Self-rated prevalence of daytime drowsiness on school days over a 1-month time interval.

‘† ‘ reference group.

Significance codes: ‘***’ ≤ 0.001 ‘**’ ≤ 0.01 ‘*’ ≤ 0.05 ‘∙ ‘ < 0.1.

Concerning sleep deprivation, we identified short sleep quotas during school nights and free nights at all three sites (Table 4a). Short sleep quotas are least frequent in Puebla and most prevalent in Campeche, with 98% of Maya participants sleeping < 9 h per night on school nights and 49% on free nights. During school nights, short sleep quotas are significantly lower among urban adolescents in Mexico City (459.42 min, SD = 39.3 min) compared to rural agricultural Totonac (490.98 min, SD = 33.69 min) and Maya (485.8 min, SD = 33 0.2 min) participants (*F*(2, 119) = 9.51, *p* = 0.0001479). After calculating sleep curtailment values for each location (Table 4b), we found that urban teenagers from Mexico City have the highest prevalence of sleep reduction during school days compared to participants from both agricultural societies in Puebla and Campeche. The sleep curtailment scores for these two sites (− 79.1 min, SD = 46.9 min and − 70.5 min, SD = 44.6 min, respectively) are significantly different from those in Mexico City (− 95.9 min, SD = 59.7 min), but not between the two rural locations (*F*(2, 133) = 3.8, *p* = 0.03). Finally, when adolescents were asked how frequently they had felt sleepy during school days over the past month (Table 4c), teenagers from Mexico City had the highest scores for daytime drowsiness and participants in Puebla the lowest.

### Bio-socio-cultural factors shaping sleep duration

#### Weeknights

**Figure 1:**
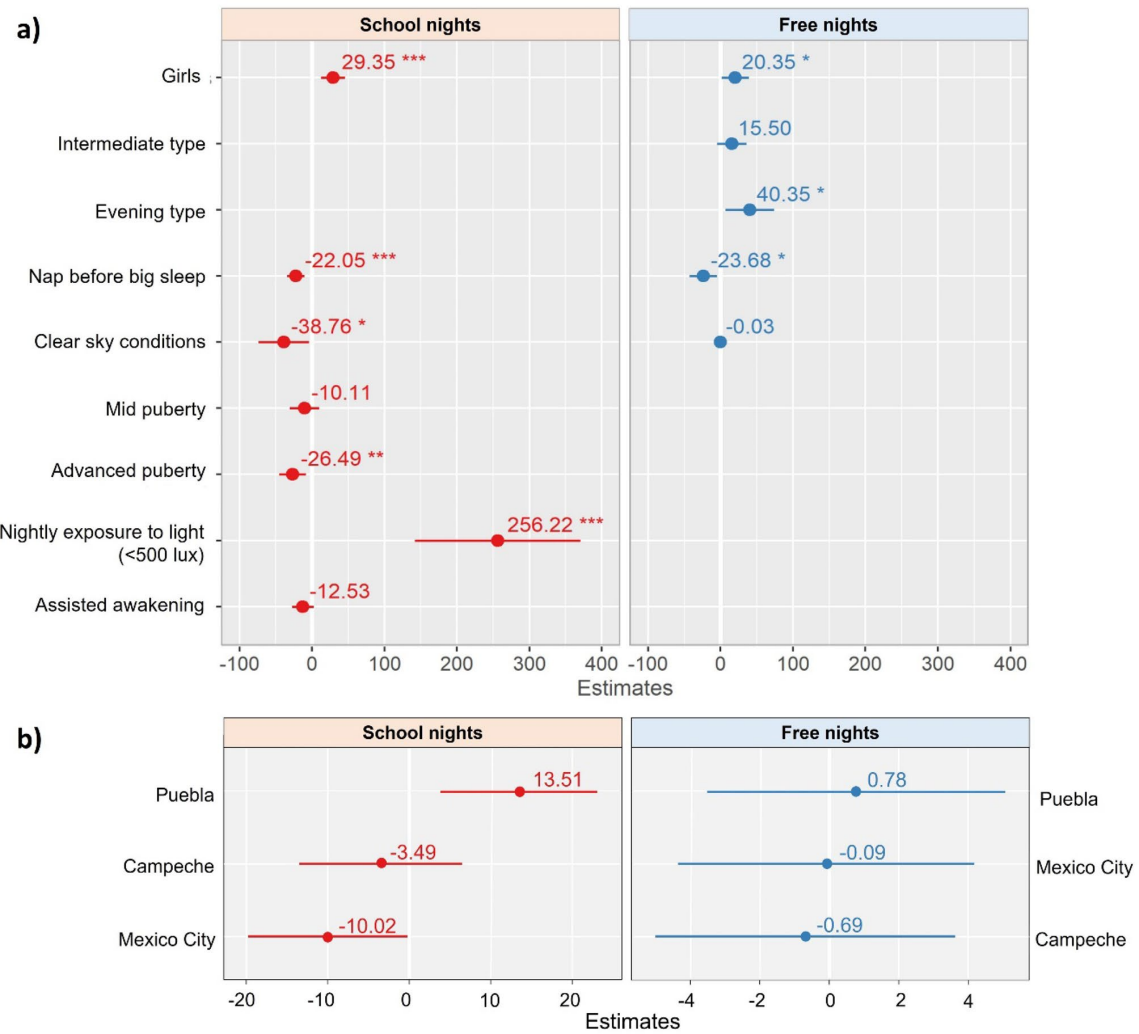
Effect sizes for all-site multilevel models. (**a**) Estimates for fixed effects (i.e., predictor variables with a constant effect on the individuals’ sleep duration). (**b**) Estimates for random effects (i.e., site-level variation on sleep duration).

The final model for school nights (Table 5) incorporates pubertal development, gender, napping before the main sleep at night, ambient light at night, clear sky conditions, and assisted awakening as predictor variables for the sleep duration variance (R^2^_Marginal/Conditional_ = 0.11/0.39; ICC_Adjusted/Conditional_ = 0.32/0.29, *df* = 595). Sleep duration is negatively influenced by pubertal development, napping before going to sleep, clear sky conditions and assisted awakening, and is positively influenced by gender and ambient light at night (Fig. 1). In contrast, the model for free nights includes gender, chronotype, napping before the main sleep, and clear sky conditions as predictor variables for sleep duration variance (R^2^_Marginal/Conditional_ = 0.04/0.23; ICC_Adjusted/Conditional_ = 0.19/0.18, *df* = 577). On free nights, sleep duration is negatively affected by napping before going to sleep, clear sky conditions, and positively affected by gender and chronotype.

**Table 5:** Bio-socio-cultural predictors of sleep duration variation on school nights and free nights across locations.

All-site final models	School nights				Free nights			
Predictor	Estimates	Confidence interval	*p*	df	Estimates	Confidence interval	*p*	df
Mid puberty	− 10.11	(− 30.22–10.00)	0.325	595				
Advanced puberty	− 26.49	(− 45.01 to − 7.98)	**0.005**	595				
Gender †	29.35	(12.83–45.88)	** < 0.001**	595	20.35	(1.79–38.90)	**0.032**	577
Nap before big sleep	− 22.05	(− 34.22 to − 9.88)	** < 0.001**	595	− 23.68	(− 42.78 to − 4.58)	**0.015**	577
Nightly exposure to light (< 500 lx)	256.22	(141.97–370.47)	** < 0.001**	595				
Clear sky conditions	− 38.76	(− 73.68 to − 3.84)	**0.03**	595	− 0.03	(− 0.08–0.02)	0.22	577
Assisted awakening	− 12.53	(− 27.54–2.47)	0.102	595				
Intermediate type					15.5	(− 4.96–35.97)	0.138	577
Evening type					40.35	(6.56–74.14)	**0.019**	577
Sites (Intercept)								
Mexico City	− 10.02				− 0.09			
Puebla	13.51				0.78			
Campeche	− 3.49				− 0.69			

Negative coefficients indicate decreased sleep durations, while positive coefficients higher sleep durations.

^†^Boys are the reference category for gender; the estimates are for girls.

Significant values are in bold.

#### Site-specific models

**Figure 2:**
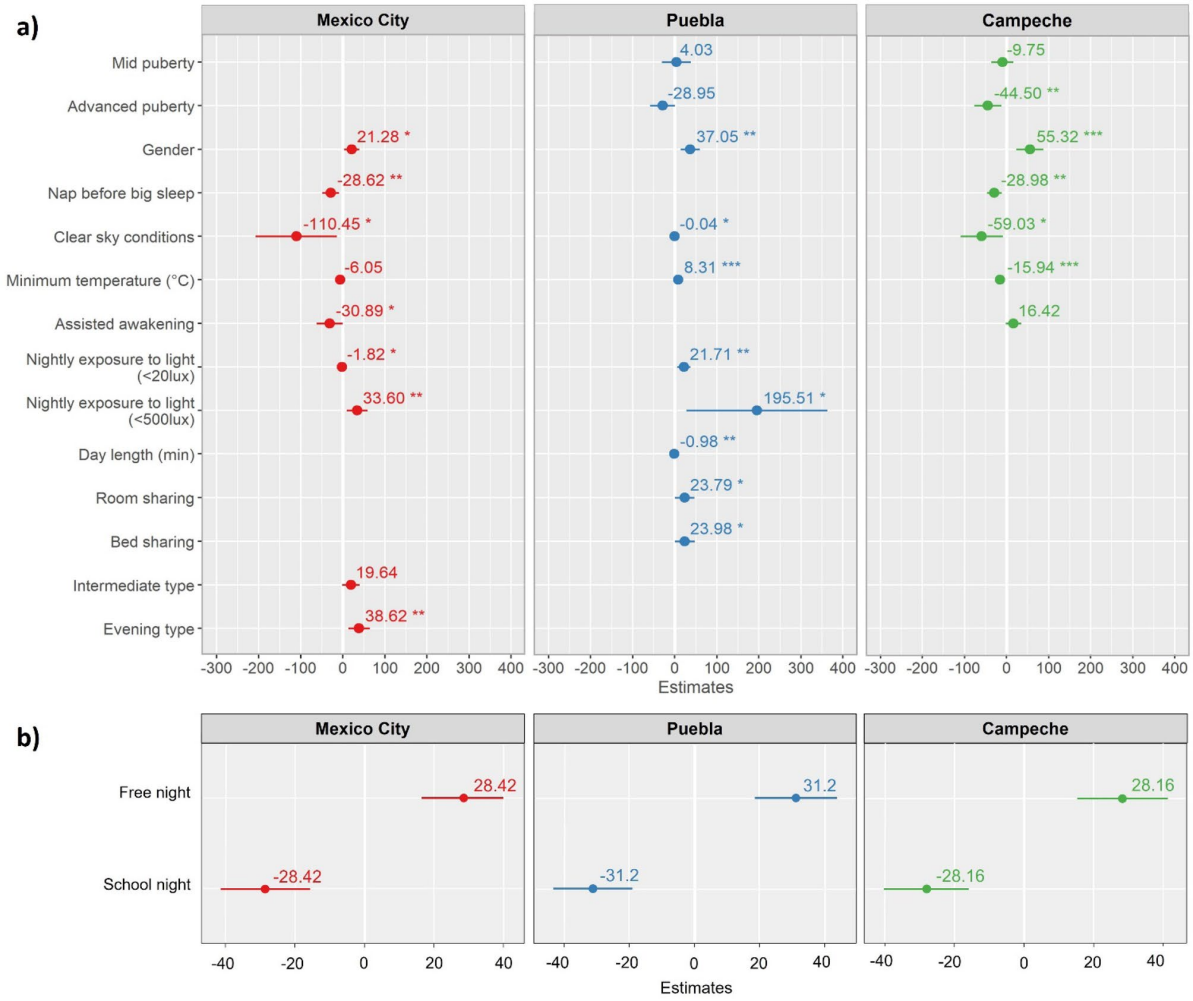
Effect sizes for site-specific multilevel models. (**a**) Estimates for fixed effects (i.e., predictor variables with a constant effect on the individuals’ sleep duration). (**b**) Estimates for random effects (i.e., weeknight-level variation on sleep duration).

The final model for Mexico City (Table 6) includes gender, chronotype, napping before the main sleep, ambient light at night, clear sky conditions, minimum temperature, and assisted awakening as predictor variables for sleep duration (R^2^_Marginal/Conditional_ = 0.11/0.35; ICC_Adjusted/Conditional_ = 0.27/0.24, *df* = 403). Gender, chronotype and standard domestic ambient light at night (between 20 and 499 Lux) positively affect sleep duration values, while naps before the main sleep, dim ambient light at night (< 20 Lux), clear sky conditions, minimum temperature and assisted awakening have a negative influence (Fig. 2). The best-fitting model for Puebla’s Totonac agricultural site comprises pubertal development, gender, nightly exposure to ambient light, clear sky conditions, minimum temperature, day length and social sleep practices as predictor variables (R^2^_Marginal/Conditional_ = 0.12/0.39; ICC_Adjusted/Conditional_ = 0.31/0.27, *df* = 404). Here, sleep duration is positively influenced by gender, ambient light at night (dim and standard domestic), minimum temperature and social sleep, while it is negatively affected by advanced puberty, clear sky conditions, and day length. Lastly, the model for Campeche’s Maya agricultural site incorporates pubertal development, gender, napping before the main sleep, clear sky conditions, minimum temperature, and assisted awakening as predictor variables (R^2^_Marginal/Conditional_ = 0.14/0.47; ICC_Adjusted/Conditional_ = 0.39/0.33, *df* = 347). Sleep duration in Campeche is positively affected by gender and assisted awakening, and negatively influenced by advanced puberty, napping, clear sky conditions, and minimum temperature.

**Table 6:** Bio-socio-cultural predictors of sleep duration values for each study location across weeknights.

Site-specific final models	Mexico city				Puebla-Totonac				Campeche-Maya			
Predictor	Estimates	Confidence interval	*p*	df	Estimates	Confidence interval	*p*	df	Estimates	Confidence interval	*p*	df
Gender	21.28	3.22–39.33	0.021	403	37.05	14.34–59.77	0.001	404	55.32	23.24–87.41	0.001	347
Intermediate type	19.64	− 1.02–40.31	0.062	403								
Evening type	38.62	13.01–64.24	0.003	403								
Nap before big sleep	− 28.62	− 48.44 to − 8.81	0.005	403					− 28.98	− 46.78 to − 11.18	0.001	347
Nightly exposure to light (< 20 lx)	− 1.82	− 3.30 to − 0.35	0.015	403	21.71	5.98–37.44	0.007	404				
Nightly exposure to light (< 500 lx)	33.6	8.70–58.50	0.008	403	195.51	27.71–363.30	0.022	404				
Clear sky conditions	− 110.45	− 207.02 to − 13.88	0.025	403	− 0.04	− 0.08 to − 0.00	0.043	404	− 59.03	− 109.34 to − 8.71	0.021	347
Minimum temperature	− 6.05	− 12.83–0.74	0.081	403	8.31	3.88–12.73	< 0.001	404	− 15.94	− 23.47 to − 8.41	< 0.001	347
Assisted awakening	− 30.89	− 61.75 to − 0.02	0.05	403					16.42	− 2.04–34.87	0.081	347
Mid puberty					4.03	− 29.99–38.05	0.816	404	− 9.75	− 35.99–16.49	0.467	347
Advanced puberty					− 28.95	− 58.30–0.40	0.053	404	− 44.5	− 76.91 to − 12.10	0.007	347
Day length (min)					− 0.98	− 1.57 to − 0.39	0.001	404				
Room sharing					23.79	0.35–47.24	0.047	404				
Bed sharing					23.98	0.54–47.43	0.045	404				
Night type (Intercept)												
School night	− 28.42				− 31.2				− 28.16			
Free night	28.42				31.2				28.16			

^†^Boys are the reference category for gender; the estimates are for girls.

## Discussion

Our main objective was to test the SJH (positing that adolescents living in “traditional”, non-industrial environments will more closely fulfil their “biological/natural” sleep requirements^25,26^) by comparing sleep deprivation among adolescents in rural and urban societies. The SJH argues that adolescent “biological/natural” sleep quotas and circadian cycles can be ascertained from free days, when sleep patterns are minimally shaped by social commitments^5,37^. Therefore, we predicted that sleep deprivation would be rare in the more rural agricultural settings of Puebla and Campeche but more frequent among participants in Mexico City. Likewise, we predicted that we would not see sleep deprivation on free days among any of the rural participants.

Our predictions were not supported, instead, we found that short sleep quotas during school nights are common in both rural agricultural settings, with over 75% of adolescents in each group sleeping < 9 h/day. Furthermore, the proportion of short sleepers in Mexico City and Campeche (the latter being the most “traditional” setting in our study), are strikingly similar (94% vs. 98%). A substantial amount of agricultural Maya adolescents in Campeche also has short sleep quotas during free days (49%), compared to Totonac (26%) and urban Mestizo (29%) participants.

Disparities in school workloads, access to screen-based devices and sunset times might explain the predominance of short sleep quotas in Mexico City and Campeche on school days. Interview data show that, compared to adolescents from agricultural areas, urban participants have greater school workloads, so they are more likely to work late at night and go to sleep later. In addition, urban adolescents have ready access to screen-based devices, increasing their chances of staying awake longer. Analogously, solar data show that Maya participants in Campeche experience longer exposure to daylight due to later sunsets than in Mexico City and Puebla, which could help delay their sleep start.

Meanwhile, on free days, waking times are not determined by fixed schedules, gaining importance in the emergence of short sleep quotas. Adolescents who have late bedtimes (possibly due to social/recreational activities paired with the absence of parent-set bedtimes, as indicated by interview and ethnographic data) and whose wake-life activities prevent them from sleeping until late morning, are more likely to have short sleep quotas (such as strict training regimes, extra-curricular activities, farming/housework or religious life, as reported by participants themselves). Therefore, our findings in Campeche, where Maya participants have the highest proportion of short sleepers during free nights, suggest that adolescent sleep in more traditional, rural settings is embedded within social commitments and expectations rather than being free from them. Possibly, past and present adolescent sleep–wake cycles in other societies (i.e., horticulturalists, hunter-gatherers, pastoralists) have not been free from social expectations either.

It should be noted that sleep deprivation during school nights is significantly less acute in agricultural adolescents, who, according to actigraphy data, sleep 26–32 min longer than urban participants. In contrast, differences in sleep deprivation are minor during free nights, ranging from 4 to 17 min between sites. Disparities in short sleep quotas on school nights might arise from differing school schedules and commuting times, as Totonac and Maya participants start classes 30 min later than urban teenagers in Mexico City and have shorter commutes.

Variability in sleep deprivation could partially explain why the proportion of participants reporting recurrent daytime sleepiness is lower among Totonac and Maya adolescents. However, the perception of drowsiness could also vary depending on the tasks socially sought and reproduced at each site. In this sense, a study by Eide and Showalter^38^ estimating the “optimal” sleep period to maximize reading and mathematics test performance in U.S.A. students aged 10–19 found that “optimal” sleep quotas changed with age and were dependent on the level of complexity of the task. Therefore, urban participants, who might be under more pressure to optimize academic performance, as indicated by interviews and ethnographic data, might require more sleep to maintain attention on complex cognitive tasks for longer. Furthermore, subjective feelings of sleepiness may be stronger in urban adolescents who perceive academic activities as dull or repetitive^15^. Still, these hypotheses remain speculative and further studies are required to test them. Additionally, in agreement with the Sleep Continuity Theory^39^ (which states that a minimum of 10 min of interrupted sleep is needed to serve a restorative function), variation in self-rated drowsiness could reflect differences in participants’ sleep efficiency (i.e., the ratio between the total time spent asleep and the total time in bed). While this matter is beyond the scope of this paper, future research will examine whether sleep efficiency is higher in both indigenous rural settings than in Mexico City.

Considering that daily sleep quotas are normally distributed in human populations, using cut off values to assess sleep sufficiency is problematic because it assumes optimal sleep is monolithic rather than variable^15,40^. Thus, we evaluated sleep curtailment during school days as a complementary approach to check sleep sufficiency^5,16^. Again, we found that sleep curtailment is pervasive in rural, agricultural adolescents, although significantly less than in Mexico City, where values are between 17 and 25 min higher than in Puebla and Campeche. As with short sleep, differences in curtailment values are likely due to specific school workloads, access to screen-based devices, sunset times, earlier school schedules, longer travel times to school and adolescents' likeliness to sleep in during free days. Altogether, our findings undermine the premises of the SJH since they suggest that, given certain socio-cultural and ecological factors, adolescents from rural, agricultural societies may also express short sleep quotas.

When we examined which specific bio-socio-cultural factors influenced nightly sleep duration in the three study sites, we found that advanced puberty had a negative impact on sleep duration during school nights, but not on free nights. This effect is most probably related to personhood associated with age rather than to sexual maturation as older teenagers usually build social bonds outside the household, gain social responsibilities (such as household or farming work) and acquire the freedom to set their own bedtimes, which in turn results in shortened sleep during school nights^4,41,42^. Arguably, such changes are decidedly more marked in subsistence rural than postindustrial urban teenagers, as indicated by interview, ethnographic and bibliographic data^43,44^. Maya and Totonac adolescents in Campeche and Puebla allocated more time than children to farming and domestic tasks to contribute to their nuclear family economy and parents. Notable, Kramer (2002) has reported that Maya girls from the same site in Campeche, increase their level of production in early adolescence (generally by age 12–13) while boys increase their work efforts later in youth (generally by age 16–17)^44^, resembling pubertal maturation. Similar age and sex differences in work efforts might also prevail in Puebla. In comparison, teenagers in Mexico City reported spending their time either learning or in leisure activities, not regarding their age, all of which could explain why the effect of advanced puberty was significant in Campeche and Puebla but not in Mexico City. Results from previous meta-analyses agree with ours inasmuch they note that sleep duration decreases with age on school days but remains the same from pre-puberty to late adolescence on free days^5,16^.

Along with age, personhood is molded by gender, a cultural variable that previous research has found to have confounding effects on adolescents' sleep duration^45^. Given that the participants of this study belong to three distinct cultural groups, we expected to encounter mixed effects of gender on sleep in our samples. Surprisingly, gender consistently influences sleep duration across all study sites and weekdays, with girls sleeping more than boys. Differences in sleep quotas could be linked to distinct gender access to screen-based devices, involvement in household activities, bedtimes routines, and time investment in getting ready for school. Notably, gender effects are larger on school days, when waking times are fixed, and also in agricultural environments, where greater social distinctions between girls and boys might exist. In this sense, our results concur with previous studies indicating that human sleep patterns are heavily shaped by the interaction of gender and socio-economic status^46,47^. Still, more research is required to clarify whether these findings could also be explained by sexual dimorphism, as some other studies have suggested^48^.

A third factor affecting participants’ sleep was chronotype, a behavioral trait that, unlike gender and sex, has been extensively investigated in relation to adolescent sleep patterns. Specifically, researchers have described a chronotype transition towards eveningness around puberty^49^ that is considered a marker of adolescent sleep–wake cycles^6^ which is itself then conducive towards sleep deprivation on school days^17^. However, we did not find a significant effect of evening chronotype on sleep quotas during school nights, but instead, found a significant positive effect during free nights. The cumulative impact of nocturnal sleep curtailment could partially explain why evening types sleep longer on free nights, presumably to recover, even though their sleep is not pointedly reduced on school nights^10^. Chronotype has the most marked effect on sleep duration in Mexico City, but not in the rural locations, where evening chronotypes are less frequent and adolescents are less prone to sleep in during free days. Similar differences in chronotype between urban and rural populations have been previously described in adolescents and adults^30,50,51^. These findings suggest that differences in social activities affect human chronotype expression and variability.

Daytime napping is another behavioral trait known for shaping nightly sleep quotas^52^. The propensity of contemporary humans to experience a mid-afternoon dip in alertness (linked to circadian oscillations of body temperature and performance) is well recognized^33,53^. Since it has been hypothesized that a napping episode during the mid-afternoon would occur under “natural”, rural conditions but would be inhibited by industrial lifestyles^22,33^, we expected to find higher napping rates in agricultural adolescents than in urban participants. Additionally, we reasoned we would observe greater nap ratios during school nights, derived from nightly short sleep quotas^54^. None of these predictions was supported by our results. Napping behavior is infrequent in all our study sites and we found no significant differences between weekdays (non-paired *t*, *p* = 0.95). This suggests, napping episodes are opportunistic and do not necessarily reflect participants alleged “sleep debt”. Even so, compared to teenagers in Campeche and Mexico City, participants in Puebla, who sleep the most during the night, have the lowest napping rates (probably because they sleep the most during the night). This distinction might be why, although napping behavior consistently shortened sleep duration across weekdays, its impact was no significant in Puebla.

Along with individual characteristics and behavioral preferences, the physical and social features of sleep environments, such as temperature, lightening and co-sleeping practices, also act as sleep modifiers^55^. For instance, thermal stress, caused by exposure to extreme temperatures, results in difficulty falling asleep and frequent awakenings^56^. Our results confirm this, given that higher temperatures in hot weather or in isolated, temperate sleeping environments (Campeche and Mexico City) give rise to shorter sleep durations but facilitate longer sleep in less shielded, colder sleeping environments (Puebla). Similarly, the effects of light on nightly sleep have been profusely addressed in the relevant literature^4,6,17,25,57^. In this regard, we presumed that daylight duration and intensity (estimated through clear sky conditions) would predict adolescent sleep duration in rural settings, but not in Mexico City, where buildings are designed for buffering external environmental cues. We confirmed that, as day length increases, participants’ sleep decreases in Puebla, but we did not find this effect in Campeche. This distinction is most probably related to seasonal differences in agricultural activities. At the time of the study, Totonac participants in Puebla had more work to do during daylight hours (i.e., maintaining, harvesting, and cleaning their crops) than Maya participants in Campeche who were engaged in weeding their lands and waiting for the harvest. We also observed that as daylight intensity increased, participants’ sleep would decrease in all sites. Daylight at high intensities is recognized for advancing or delaying sleep depending on whether the individual was exposed to morning or evening/night light, respectively^57^. Although we cannot infer the exact time of participants' exposure to daylight, we can assume that on non-cloudy, rain-free days, adolescents could perform diverse outdoor social and physical activities which would have inhibited sleep propensity and negatively affected sleep duration.

We hypothesized that the exposure to artificial lights at night would shorten sleep during school days, with a marked effect across sites given previous studies have reported light exposure is associated with shorter sleep durations^17,23,58,59^. Surprisingly, exposure to light at night has mixed effects on sleep quotas depending on its intensity and social context. For example, while standard domestic ambient light at night (< 500 Lux) increases sleep duration on school nights, dim ambient light at night (< 20 Lux) shortens sleep quotas in Mexico City and increases them in Puebla. Moreover, nocturnal illumination does not affect sleep in Campeche, potentially because of participants' tendency to sleep in dark environments and their limited access to screen-based devices, mobile/cell phone coverage and the Internet.

Bringing adolescent sleep into its socio-cultural context together with its emotional dynamics is essential for understanding our contrasting results. Urban and rural participants would commonly report falling asleep under lightened conditions, either because they were watching images on their TV/phone/tablet or because those with whom they shared rooms would keep the lights on until late at night. In particular, agricultural Totonac participants in Puebla emphasized that having a light on while sleeping made them feel safe at night, a dangerous period when natural and supernatural characters (e.g., human and non-human predators, sorcerers, evil spirits or dead people) lurk in the shadows. Hence, adolescents whose sleep was exposed to < 500 Lux on school nights probably began sleeping before domestic lights went off due to exhaustion (i.e., strong sleep pressure) and the lights’ inhibitory effect on arousing emotions, two factors that would promote sleep. On the other hand, lights < 20 Lux mainly coming from electronic media or undesired luminous pollution in Mexico City would presumably elicit arousal among urban participants, thus shortening their sleep. In contrast, exposure to < 20 Lux resulting from moonlight, streetlights and domestic lights in Puebla would have helped to inhibit arousal among Totonac adolescents, facilitating longer sleep quotas.

Assisted awakening and social sleep practices provide further evidence of the interrelationship of adolescent sleep and its psycho-socio-cultural context. Even though assisted awakening tends to shorten sleep duration on school nights, an opposite trend is observed in Campeche. It is possible that, as Maya participants are generally awakened by the sun, dogs barking, roosters or noise of other household members, they would only have needed assistance to wake up when all other awakening cues had failed. This would mean that, contrary to what happened in Mexico City or Puebla, participants who need assistance for awakening in Campeche end up waking later and, thus, sleeping longer. Lastly, social sleep, commonly practiced in agricultural settings, has a marked positive effect on sleep duration in Puebla, while tending to shorten sleep in Mexico City and Campeche. As above, the participants’ cognitive-emotional state might underlie the distinct effects of social sleep^29^. Unlike urban and Maya participants, adolescents in Puebla would have found sleeping with others comforting and reassuring, inhibiting alertness and enabling prolonged sleep quotas.

Taken together, our results suggest that the prevalence of adolescent short sleep quotas is not less in rural, agricultural and more traditional environments than in post-industrial urban societies. These findings bring into question current assumptions about sufficient sleep and how adolescents slept before the modern era. Contrary to the SJH, reduced adolescent sleep durations might have been a constant in our species’ evolutionary history where individuals weighed the costs of reduced sleep against the benefits to them or their group of economic, social, reproductive or rearing waking-life activities^22,60^. Consequently, we advocate further research to delve into the relationship between sleep and health outcomes in non-clinic, rural settings to better understand sleep's role in our evolutionary history.

Additionally, our study highlights the influence of ontogenetic development on the expression of human chronotypes, where a combination of genetic and epigenetic factors (potentially modulated by bio-socio-cultural factors such as photoperiod, temperature, developmental stressors, lifestyle, or parental involvement in offspring sleep) give rise to distinct circadian rhythms from prenatal development to old age^61^. Notably, despite the role of circadian rhythms in the maintenance of cognition, behavior and mental wellbeing, the study of epigenetic mechanisms is recent and mainly focused on non-human models^62^. Thus, we stress the need to incorporate a developmental approach to the study of infant and adolescent sleep to shed light on the epigenetic regulation of human biological rhythms and its short- and long-term consequences in health.

This research is subject to limitations. Firstly, our sample size was small, limited by convenience sampling in rural and urban settings. Secondly, we lacked longitudinal data, critical for drawing comparisons between different age groups and identifying sleep developmental trajectories. Additionally, we could not incorporate data on seasonal variations in diet, energy expenditure and allocation, which might impact sleep traits. Thirdly, the Morningness-Eveningness Reduced Scale (MERS), employed for assessing the phase preferences of participants over a 24-h day, requires individuals to structure time in hours, minutes, and seconds, and not as a function of socio-ecological cues (such as sunrise, sunset, meal times, non-human animal behavior, radio or TV programming, etc.). Although most of the study participants were familiar with “modern” uses of time, some were not, which might have reduced MERS accuracy. Similarly, the PDS, a self-reported instrument for evaluating pubertal status, may fail to reflect the precise developmental stage of adolescents compared to Tanner stages.

This study provides novel evidence about variation in adolescent sleep quotas through the examination of sleep in one post-industrial, densely urban and two agricultural indigenous sites in Mexico. We advocate further sleep research employing an ecological, cross-cultural perspective to broaden our understanding of human sleep development, variability, health and evolution. Such an approach could help guide future research agendas that translate into more equitable and effective health policies and practices for child and adolescent wellbeing.

## Methods

### Geographical locations and participants

We worked with 163 participants (females = 78, males = 85) from three sites in Mexico: one densely populated, postindustrial metropolis (Mexico City, n = 67), one indigenous agricultural Totonac town (San Juan Ozelonacaxtla, Puebla, n = 51), and one subsistence farming Maya village (Xculoc, Campeche, n = 45) (Supplementary Fig. 1) Adolescents between 11 and 16 years old (mean age 13.7, SD ± 1.21) recruited from local schools participated in the study over 10 days comprising four non-school (free) days and six school days. The study was conducted in 2019 from February 1st to April 8th, in Mexico City, from May 31st to July 5th, in Campeche, and from September 6th to November 11th, in Puebla. Each site is characterized in Table 2 (day length, precipitation and temperature details are in Supplementary Table 1).

Participants and their parents provided written signed informed consent after obtaining a verbal explanation of the research and reading the study consent form. Consent in Maya and Totonaku was obtained with the support of local assistants fluent in either Highland Totonaku and Spanish or Peninsular Maya and Spanish. The study protocol was approved by the Ethics Committee of the Department of Anthropology at Durham University. All procedures were in accordance with the Declaration of Helsinki, complying with guidelines established for the execution of health research projects in Mexico by the Mexican General Health Regulation on Health Research Matters and the Mexican Official Standard NOM-012-SSA3-2012.

### Instruments

We employed actigraphy devices, sleep diaries, semi-structured interviews, ethnographic observations and two standardized questionnaires to assemble data regarding adolescent sleep. In particular, we measured and characterized participants' sleep variables (see Table 3.b for sleep variables definitions), pubertal maturation, daily activities (i.e., schooling and/or work efforts), and environmental factors (i.e., access to electricity and electronic devices, exposure to seasonal thermal and luminous variations, bedding characteristics, solitary or social sleeping practices).

#### Actigraphy

Unlike polysomnography (PSG), which measures sleep–wake patterns through neuronal activity, actigraphy infers being wake or sleep based on the presence or absence of movement^45^. Although PSG remains the gold standard for quantifying sleep, actigraphy provides a more objective sleep measure than sleep diaries or questionnaires and has proven to be useful at quantifying rest-activity rhythms, sleep timing, sleep duration, nighttime wake-bouts and daytime napping^20,21,23,25,59,63^. As a non-invasive method is particularly suitable for field-based studies since it is cheaper than PSG, easy to use in ambulatory populations and causes minimal disruption to sleep^24,64^. Additionally, actigraphy devices can capture data continuously over long periods in the context of subjects’ everyday environments and activities^24,45^.

A total of 147 participants wore a Motionlogger Micro Watch (Ambulatory Monitoring Inc., US) on their non-dominant wrist during 10 continuous days for 24 h to estimate sleep characteristics and ambient light. The actigraphs were set for 1-min epochs in the Zero Crossing Mode (ZCM). Participants were asked to mark the start of any sleep event during the study using the event logger button. As two teenagers did not have valid actigraphy data due to watch malfunctions, our final sample was reduced to 145 participants (Mexico City, n = 50; Puebla, n = 51; Campeche, n = 44), with 72 being females.

We assembled 1,405 sleep observations, of which 618 were free nights and 787 school nights. The actigraphy records were analyzed with the ActionW 2.7 software employing the Sadeh algorithm to characterize the sleep–wake behaviour of adolescents. As recommended, we used sleep diaries to validate further the accuracy of sleep scores generated by the software^23,65^. When encountering a nap episode, missing data (caused by a watch malfunction or off-wrist period) or additional information regarding the participant's sleep–wake behavior derived from the sleep diary, the analyst edited the scores manually. This approach allowed us to identify “false” sleep-like or wake-like entries^64^.

#### Sleep diaries and questionnaires

Sleep–wake behavior and drowsiness during the day were captured using a customized, simplified version of the Pediatric Daytime Sleepiness Scale (PDSS) and the Consensus Sleep Diary^66^. Sleep diaries employed colors, images and scales to encourage completion by adolescents with limited literacy. All participants were asked to complete their 10-day sleep diary before and after each nightly sleep.

To obtain information about the adolescents' chronotype and pubertal maturation, every participant responded to two standardized questionnaires: the Morningness-Eveningness Reduced Scale (MERS)^67^ and the Pubertal Development Scale (PDS)^68^. Both questionnaires were verbally explained to participants and any questions answered. Together with sleep diaries, data from MERS helped us validate further the accuracy of actigraphy measurements and overcome technical hurdles, such as watch removal periods. The PDS, a non-invasive, self-report instrument was used to assess pubertal status.

#### Interviews and ethnographic observations

We used semi-structured interviews (lasting 45 min on average) to capture information about the sleeping environment of every participant. Specifically, questions were aimed to assess: (1) ongoing sleep–wake patterns and sleep quality, (2) cultural ideas and practices sorrounding sleep, (3) characteristics and cultural settings in which adolescents slept, and (4) access to electricity and/or electronic devices. Interviews were based on Grandner et al.^70^ and included items from the Pittsburgh Sleep Quality Index (PSQI)^69,70^. Ethnographic observations were made in and outside the schools to explore social roles further, peer relationships, attitudes towards sleep and daily lives of adolescents. A field diary was employed to record observations, daily activities, notes about interviews, casual conversations and comments concerning research development.

#### Meteorological and solar variables

Geographical coordinates for the three sites were utilized with the NASA Langley Research Center (LaRc) POWER Project (https://power.larc.nasa.gov/) to attain daily data on surface pressure, precipitation, humidity, sky insolation incident, temperature, and the Insolation Clearness Index. Additionally, based on Iqbal (1983), daily data on the times of sunrise, sunset and day length were obtained using solar geometry^71^.

### Data analyses

To examine whether adolescents in rural agricultural societies experience sleep deprivation, we focused on the variation of participants’ sleep duration (i.e., the interval between going to sleep, and the time one rising from of bed/hammock/sleep surface) and total sleep duration (i.e., the total amount of time spent in bed per day, including daytime naps). In particular, we employed two of the most utilized approaches to evaluate sleep sufficiency: (1) to assess if participants slept a minimum of 9 h per night as recommended by USA advisory bodies^12,14,26^, and (2) to evaluate sleep curtailment during school days measured as the difference in adolescents’ mean values for total sleep duration (TSD) between school nights and free nights^4,5,72^. The first approach is based on the findings of two seminal studies investigating the effect of different sleep durations on adolescent daytime sleepiness^73,74^; the latter is supported by two meta-analyses reporting that TSD decreases with age only when recordings take place on school nights, remaining constant and sufficient on free nights from pre to late adolescence^5,16^. It is noteworthy that these studies were performed on urban populations, predominantly from rich, industrialized countries.

Descriptive statistics characterized daytime napping, sleep timing and sleep duration in our sample population. Then, we utilized an unpaired t-test to look for significant differences in adolescent sleep duration values between school and free nights, and paired t-tests to assess whether participants’ sleep duration and TSD values had significant differences on school nights and free nights. Next, we ran two ANOVAs to check if there were significant differences in sleep duration and TSD values between our three sites. We subtracted each participant’s mean TSD during school days from their mean TSD during free days to gauge sleep curtailment on school days and then used an ANOVA to determine if the resultant values differed significantly between the three sites. All tests were two-tailed. Finally, we ran Benjamini–Hochberg post hoc tests to determine specific differences between sites.

To investigate the influence of sexual maturation, natural and artificial light, and social sleep practices on adolescent sleep duration on school nights and free nights, we fitted two multilevel models with the *lme4* R-package^75^. We incorporated "subject" and "site" as random effects to control for repeated measurements of individuals who were nested within three distinct sites. Furthermore, we fitted three site-specific models to unravel the specific influences of our predictor variables on the adolescent sleep duration in each site. In these models, we incorporated "subject" and "condition" (i.e., school nights and free nights) as random effects to control for repeated measurements of individuals who were nested within two different conditions.

All our models tested pubertal maturation (typified as pre/early, mid-, and advanced puberty), chronotype (scored as morning, neither and evening type), gender (female/male), daytime napping, nightly light exposure (the percentage lux count epochs in relation to participant’s sleep duration), nightly exposure to screen-based devices (NESDI-index of the number of devices being handled after 8 pm), day length, clear sky conditions (assessed via the Insolation Clearness Index, with values close to 1 under clear, sunny conditions and to 0 under cloudy conditions), minimum temperature, assisted awakening (the usage of an external agent, alarm or person, to wake up), and social sleep practices (the practice of room-sharing or bed-sharing) as predictor variables (resulting full models available in Supplemental Tables 2,3). When fitting and selecting our final models, we employed the *MuMin* R-package^76^ using the Akaike Information Criteria (AIC) and the Bayesian Information Criterion (BIC). The residuals of all the best-fitting models were normally distributed. All statistical analyses were conducted with significance levels set at 0.05 using R software version 3.6.3. Statistical inferences were established using confidence intervals and *p*-values.

## Supplementary Information

Supplementary Information.

## Data Availability

The datasets generated and analysed during the current study are publicly available in the Durham Research Online DATAsets Archive (DRO-DATA) under the name Daily adolescent sleep, Mexico 2019_Dataset 10.15128/r16682x402c.
