# Supplementary material for: Sleep deprivation among adolescents in urban and indigenous-rural Mexican communities
Source: Sci Rep. 2023 Jan 19;13:1058. doi: 10.1038/s41598-023-28330-8 (PMC9852252; doi:10.1038/s41598-023-28330-8)
Supplement: Supplementary file 1 — Supplementary Information. [file 41598_2023_28330_MOESM1_ESM.docx]

**Supplementary Figure 1. Study locations. Maps edited by Andrea Silva-Caballero based on: a) INEGI, Municipal geostatistical framework, 2016, b) Karen Kramer and Russell Graves, 2017, c) Google Earth and Ludovico Núñez, 2019.**


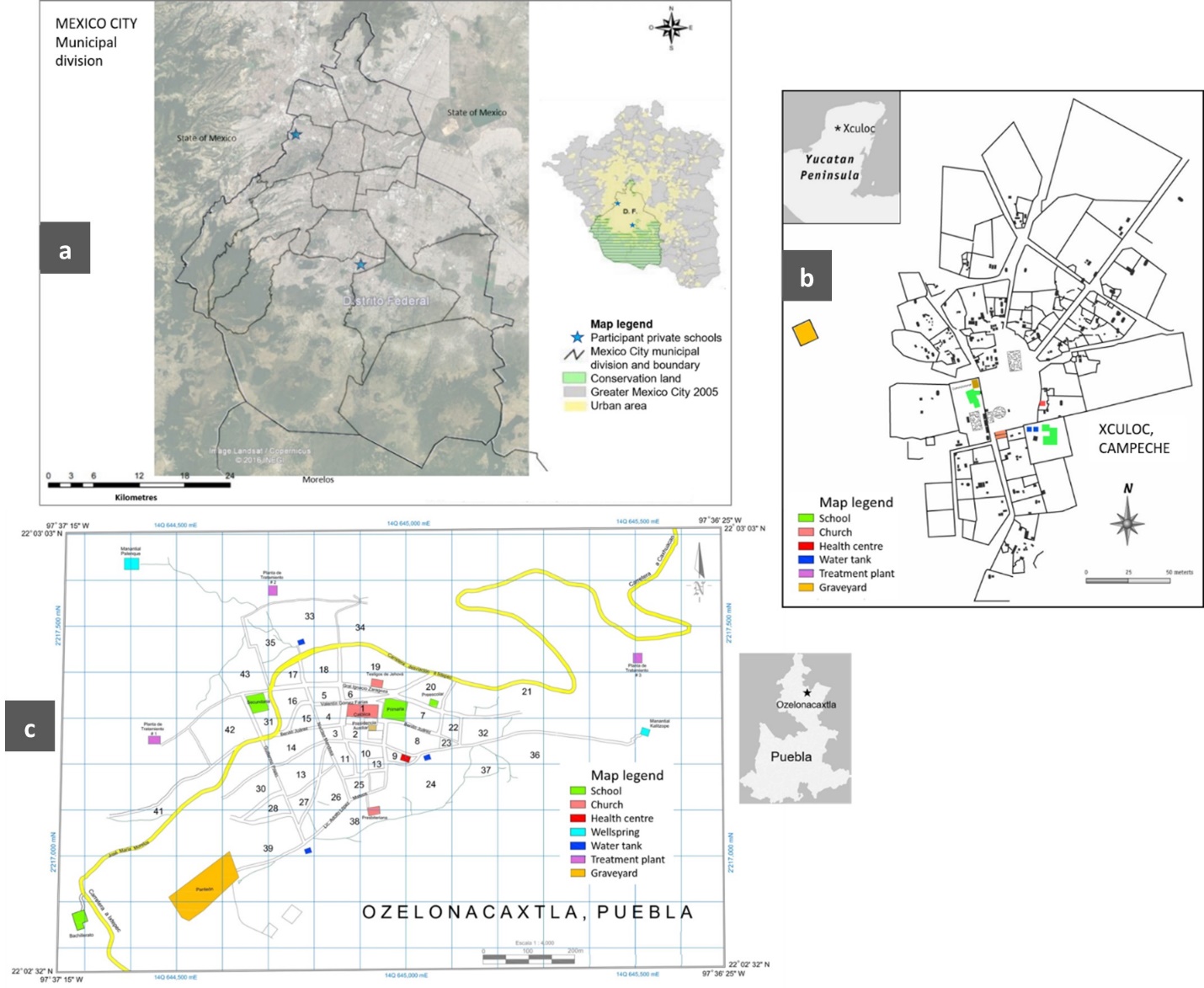


**Supplementary Table 1. Day length, precipitation and temperature in Mexico City, Campeche and Puebla**

| **Site** | **Month** | **Sunrise** | **Sunset** | **Day Length** | **Precipitation (mm/day)** | **Maximum Temp (ºC)** | **Minimum Temp (ºC)** | **Average Temp (ºC)** | **Insolation Clearness Index** |
| --- | --- | --- | --- | --- | --- | --- | --- | --- | --- |
| Mexico City | February | 7:09:06 | 18:31:37 | 11:22:31 | 0.17 | 24.97 | 8.21 | 15.64 | 0.65 |
|  | March | 6:48:32 | 18:42:47 | 11:54:14 | 0.21 | 27.23 | 9.1 | 17.42 | 0.68 |
|  | April | 7:11:10 | 19:38:30 | 12:27:19 | 0.3 | 28.27 | 9.97 | 18.44 | 0.64 |
|  | *Average* | 7:07:26 | 19:10:59 | 12:03:32 | 0.23 | 27.18 | 9.22 | 17.40 | 0.67 |
| Xculoc, Bolonchen, Campeche | May | 6:27:08 | 19:24:25 | 12:57:16 | 2.63 | 37.33 | 24.98 | 30.65 | 0.61 |
|  | Jun | 6:23:32 | 19:35:32 | 13:12:00 | 7.48 | 34.74 | 24.64 | 29.21 | 0.56 |
|  | July | 6:31:59 | 19:37:50 | 13:05:50 | 5.2 | 33.47 | 23.62 | 28.11 | 0.62 |
|  | *Average* | 6:31:51 | 19:45:27 | 13:13:35 | 5.13 | 35.57 | 24.68 | 29.65 | 0.60 |
| SJO, Huehuetla, Puebla | September | 7:21:02 | 19:30:10 | 12:09:07 | 4.19 | 28.56 | 22.67 | 18.28 | 0.51 |
|  | October | 7:28:49 | 19:03:41 | 11:34:52 | 8 | 25.39 | 20.65 | 16.99 | 0.5 |
|  | November | 6:49:31 | 17:54:12 | 11:04:41 | 2.04 | 22.83 | 17.65 | 13.78 | 0.47 |
|  | *Average* | 7:18:07 | 19:02:04 | 11:43:57 | 4.83 | 25.88 | 16.54 | 20.55 | 0.51 |

**Supplemental Table 2. Full multilevel models for school nights and free nights**

| **All-site full models** | **School nights** | | | | **Free nights** | | | |
| --- | --- | --- | --- | --- | --- | --- | --- | --- |
| *Predictor* | *Estimates* | *Confidence interval* | *p* | *df* | *Estimates* | *Confidence interval* | *p* | *df* |
| Mid puberty | -9.6 | (-29.80 – 10.61) | 0.352 | 593 | -5.85 | (-35.22 – 23.51) | 0.696 | 573 |
| Advanced puberty | -26.74 | (-45.16 – -8.32) | **0.004** | 593 | -17.95 | (-44.48 – 8.58) | 0.185 | 573 |
| Gender † | 30.48 | (13.75 – 47.20) | **<0.001** | 593 | 26.46 | (2.90 – 50.02) | **0.028** | 573 |
| Intermediate chronotype | -4.42 | (-18.87 – 10.03) | 0.549 | 593 | 11.67 | (-9.12 – 32.47) | 0.271 | 573 |
| Evening chronotype | -13.95 | (-38.50 – 10.59) | 0.265 | 593 | 39.55 | (5.73 – 73.37) | **0.022** | 573 |
| Nap before big sleep | -22.08 | (-34.24 – -9.91) | **<0.001** | 593 | -22.52 | (-41.63 – -3.41) | **0.021** | 573 |
| Nightly exposure to light (<500 lux) | 254.54 | (140.25 – 368.82) | **<0.001** | 593 | 6.41 | (-7.79 – 20.60) | 0.376 | 573 |
| Clear sky conditions | -38.88 | (-73.79 – -3.98) | **0.029** | 593 | -0.03 | (-0.08 – 0.02) | 0.248 | 573 |
| Assisted awakening | -12.31 | (-27.26 – 2.64) | 0.107 | 593 | 11.96 | (-13.24 – 37.16) | 0.352 | 573 |
| *Sites (Intercept)* |  |  |  |  |  |  |  |  |
| Mexico City | -9.00 |  |  |  | -0.14 |  |  |  |
| Puebla | 13.48 |  |  |  | 7.58 |  |  |  |
| Campeche | -4.47 |  |  |  | -7.49 |  |  |  |
| † Boys are the reference category for gender; the estimates are for girls | | | | | | | | |

**Supplemental Table 3. Full multilevel models for Mexico City, Puebla and Campeche**

| **Site-specific full models Mexico City** | | | | | | | **Puebla-Totonac** | | | | | | | **Campeche-Maya** | | | | | | | | | | | | |  |
| --- | --- | --- | --- | --- | --- | --- | --- | --- | --- | --- | --- | --- | --- | --- | --- | --- | --- | --- | --- | --- | --- | --- | --- | --- | --- | --- | --- |
| *Predictor* | *Estimates* | *Confidence interval* | | *p* | *df* | | | | *Estimates* | *Confidence interval* | | *p* | *df* | | | *Estimates* | *Confidence interval* | | | | *p* | | | *df* | | |  |
| Mid puberty | 0.6 | (-27.31 – 28.52) | 0.966 | | | 395 | | 6.23 | | (-29.76 – 42.22) | 0.734 | | 398 | | -4.73 | | (-32.84 – 23.37) | 0.741 | | | | 337 | | | |  |  |
| Advanced puberty | 9.38 | (-16.60 – 35.36) | 0.479 | | | 395 | | -28.07 | | (-57.87 – 1.74) | 0.065 | | 398 | | -39.65 | | (-73.92 – -5.38) | **0.023** | | | | 337 | | | |  |  |
| Gender † | 11.35 | (-11.11 – 33.82) | 0.322 | | | 395 | | 35.58 | | (12.10 – 59.06) | **0.003** | | 398 | | 44.02 | | (9.48 – 78.55) | **0.012** | | | | 337 | | | |  |  |
| Intermediate chronotype | 28.52 | (6.19 – 50.85) | **0.012** | | | 395 | | -4.31 | | (-27.43 – 18.81) | 0.715 | | 398 | | -8.67 | | (-31.37 – 14.03) | 0.454 | | | | 337 | | | |  |  |
| Evening chronotype | 49.42 | (22.26 – 76.59) | **<0.001** | | | 395 | | -39.09 | | (-88.73 – 10.54) | 0.123 | | 398 | | -0.79 | | (-74.27 – 72.69) | 0.983 | | | | 337 | | | |  |  |
| Nap before big sleep | -27.9 | (-47.68 – -8.12) | **0.006** | | | 395 | | -11.4 | | (-31.26 – 8.46) | 0.261 | | 398 | | -26.24 | | (-44.36 – -8.12) | **0.005** | | | | 337 | | | |  |  |
| NESDi (1) | -7.15 | (-31.59 – 17.29) | 0.566 | | | 395 | | 12.61 | | (-14.76 – 39.98) | **0.367** | | 398 | | -10.91 | | (-35.83 – 14.01) | 0.391 | | | | 337 | | | |  |  |
| NESDi (2) | -21.6 | (-51.09 – 7.89) | 0.151 | | | 395 | | 12.04 | | (-14.83 – 38.91) | 0.38 | | 398 | | -10.96 | | (-37.60 – 15.67) | 0.42 | | | | 337 | | | |  |  |
| NESDi (3+) | -38.27 | (-76.21 – -0.34) | **0.048** | | | 395 | | N/A | | N/A | N/A | | N/A | | -42.23 | | (-95.41 – 10.95) | 0.12 | | | | 337 | | | |  |  |
| Nightly exposure to light (<20 lux) | -1.71 | (-3.18 – -0.24) | **0.023** | | | 395 | | 21.19 | | (5.43 – 36.94) | **0.008** | | 398 | | -0.28 | | (-2.26 – 1.70) | 0.785 | | | | 337 | | | |  |  |
| Nightly exposure to light (<500 lux) | 35.92 | (10.99 – 60.85) | **0.005** | | | 395 | | 186.86 | | (18.75 – 354.9)7 | **0.029** | | 398 | | 1.62 | | (-10.62 – 13.87) | 0.795 | | | | 337 | | | |  |  |
| Day length (min) | 0.28 | (-0.18 – 0.75) | 0.229 | | | 395 | | -0.94 | | (-1.53 – -0.35) | **0.002** | | 398 | | 1.05 | | (-3.82 – 5.93) | 0.672 | | | | 337 | | | |  |  |
| Clear sky conditions | -109.96 | (-206.71 – -13.21) | **0.026** | | | 395 | | -0.04 | | (-0.08 – -0.00) | **0.039** | | 398 | | -64.59 | | (-119.01 – -10.17) | **0.02** | | | | 337 | | | |  |  |
| Minimum temperature | -7.13 | (-14.14 – -0.13) | **0.046** | | | 395 | | 8.14 | | (3.69 – 12.58) | **<0.001** | | 398 | | -15.2 | | (-22.86 – -7.53) | **<0.001** | | | | 337 | | | |  |  |
| Room sharing | -19.43 | (-39.84 – 0.99) | 0.062 | | | 395 | | 25.05 | | (2.08 – 48.01) | **0.033** | | 398 | | -22.22 | | (-54.04 – 9.59) | 0.171 | | | | 337 | | | |  |  |
| Bed sharing | -1.65 | (-47.40 – 44.09) | 0.944 | | | 395 | | 18.04 | | (-6.71 – 42.79) | 0.153 | | 398 | | -45.45 | | (-86.57 – -4.32) | **0.03** | | | | 337 | | | |  |  |
| Assisted awakening | -26.16 | (-57.33 – 5.00) | 0.1 | | | 395 | | -16.73 | | (-40.90 – 7.43) | 0.175 | | 398 | | 20.26 | | (1.91 – 38.62) | **0.03** | | | | 337 | | | |  |  |
| *Night type (Intercept)* | |  | |  |  | | |  | |  |  | |  | |  | |  | | |  | | |  | | | |  |
| School night | -30.01 |  | |  |  | | | -25.76 | |  |  | |  | | -28.39 | |  | | |  | | |  | | | |  |
| Free night | 30.01 |  | |  |  | | | 25.76 | |  |  | |  | | 28.39 | |  | |  | | |  | | |  |  |  |
| † Boys are the reference category for gender; the estimates are for girls | | | | | | | | | | | | | | | | | | | | | | | | | | |  |
